# SLC39A4 expression is associated with enhanced cell migration, cisplatin resistance, and poor survival in non-small cell lung cancer

**DOI:** 10.1038/s41598-017-07830-4

**Published:** 2017-08-03

**Authors:** Dong-ming Wu, Teng Liu, Shi-hua Deng, Rong Han, Ying Xu

**Affiliations:** Clinical Laboratory, The First Affiliated Hospital of Chengdu Medical College, Chengdu, Sichuan 610041 P.R. China

## Abstract

The zinc transporter SLC39A4 influences epithelial cell morphology and migration in various cancers; however, its role in regulating cell invasion and chemotherapeutic resistance in human lung cancer is not yet clear. Here, integrated analysis of gene expression in non-small cell lung cancer revealed that SLC39A4 expression is significantly correlated with increased tumour size and regional lymph node spread, as well as shorter overall survival (OS) and disease-free survival (DFS). SLC39A4 silencing by lentivirus-mediated shRNA blocked human lung cancer cell epithelial-mesenchymal transition and metastasis *in vitro* and *in vivo*, respectively. Moreover, SLC39A4 knockdown enhanced cancer cell sensitivity to cisplatin-induced death by inhibiting stemness in lung cancer cells. Collectively, these data suggest that SLC39A4 may be a novel therapeutic target and predictive marker of tumour metastasis in non-small cell lung cancer.

## Introduction

Recent evidence indicates that the zinc transporter SLC39A4 promotes cell invasion and apoptotic resistance in pancreatic cancer^1, 2^, glioma^3, 4^, and prostate carcinoma^5^ through multiple mechanisms such as IL6/STAT3 pathway activation^6^ or regulation of protein expression^7^. However, although these findings indicate that SLC39A4 plays an important role in cancer cell migration and survival, the molecular mechanisms underlying these processes require further elucidation.

The epithelial-mesenchymal transition (EMT) drives polarized, immotile epithelial cells to acquire apolar, highly migratory fibroblastoid-like features. This process is indispensable for organogenesis, tissue remodelling, and wound healing^8^, but it is also a means for cancer metastasis. The functional significance of EMT in cancer metastasis is based on the observation that the acquisition of mesenchymal markers such as vimentin or fibroblast-specific protein 1 (FSP1) and loss of cell adhesion molecules such as E-cadherin in epithelial carcinoma cells is associated with increased metastatic potential^9, 10^. Moreover, the EMT also plays a dominant role in the initial stage of cancer cell migration, where epithelial-derived tumour cells obtain a propensity for migration and deformation that enables their invasion into the basement membrane^11^. Concurrently, the EMT can induce stem cell-like characteristics in cancer cells, enhancing their resistance to chemotherapy^12^. Nevertheless, the participation of SLC39A4 in EMT, stemness, and the chemotherapeutic resistance of cancer cells requires further research.

Lung cancer is one of the most common malignancies worldwide and the leading cause of cancer-related death in men and women^13, 14^. Tumours are often classified into two main subtypes, i.e., small cell lung cancer (SCLC) and non-small cell lung cancer (NSCLC), based on the size and appearance of cancer cells under the microscope. NSCLC accounts for the majority of lung cancers and is generally characterized by regional lymph node metastasis and hematogenous spread^15^. Lung cancer development and progression are complex processes that involve multiple causes, including environmental factors, long-term exposure to radioactive materials and their derivatives, and genetic mutations^16^. Metastatic lung cancer cells are usually resistant to radiation and chemotherapy, greatly limiting the prognosis of these patients^17^; thus, the identification of metastasis-related biomarkers in lung cancer is particularly relevant for the development of precision therapies^18^.

The growing prevalence of high-throughput genomics has generated large amounts of data pertinent to cancer research^19^, leading to the formation of multiple cancer-specific gene expression databases, such as the Gene Expression Omnibus (GEO) and the Cancer Genome Atlas (TCGA)^20, 21^. To date, little is known about the clinical significance and function of SLC39A4 in NSCLC pathogenesis; therefore, this study aimed to investigate its relevance in meta-analyses of data obtained from GEO and TCGA. We subsequently confirmed our results by tissue arrays, SLC39A4 functional studies in cultured cells and animal models.

Here, we showed that SLC39A4 upregulation is a prognostic indicator of NSCLC. Cell-based assays also demonstrated an important role for SLC39A4 in the diminished migratory potential, stemness, and chemoresistance of A549 lung cancer cells both *in vitro* and *in vivo*. Collectively, this research suggests that SLC39A4 may be a novel therapeutic target in NSCLC.

## Results

### SLC39A4 is an independent predictor of NSCLC

**Figure 1:**
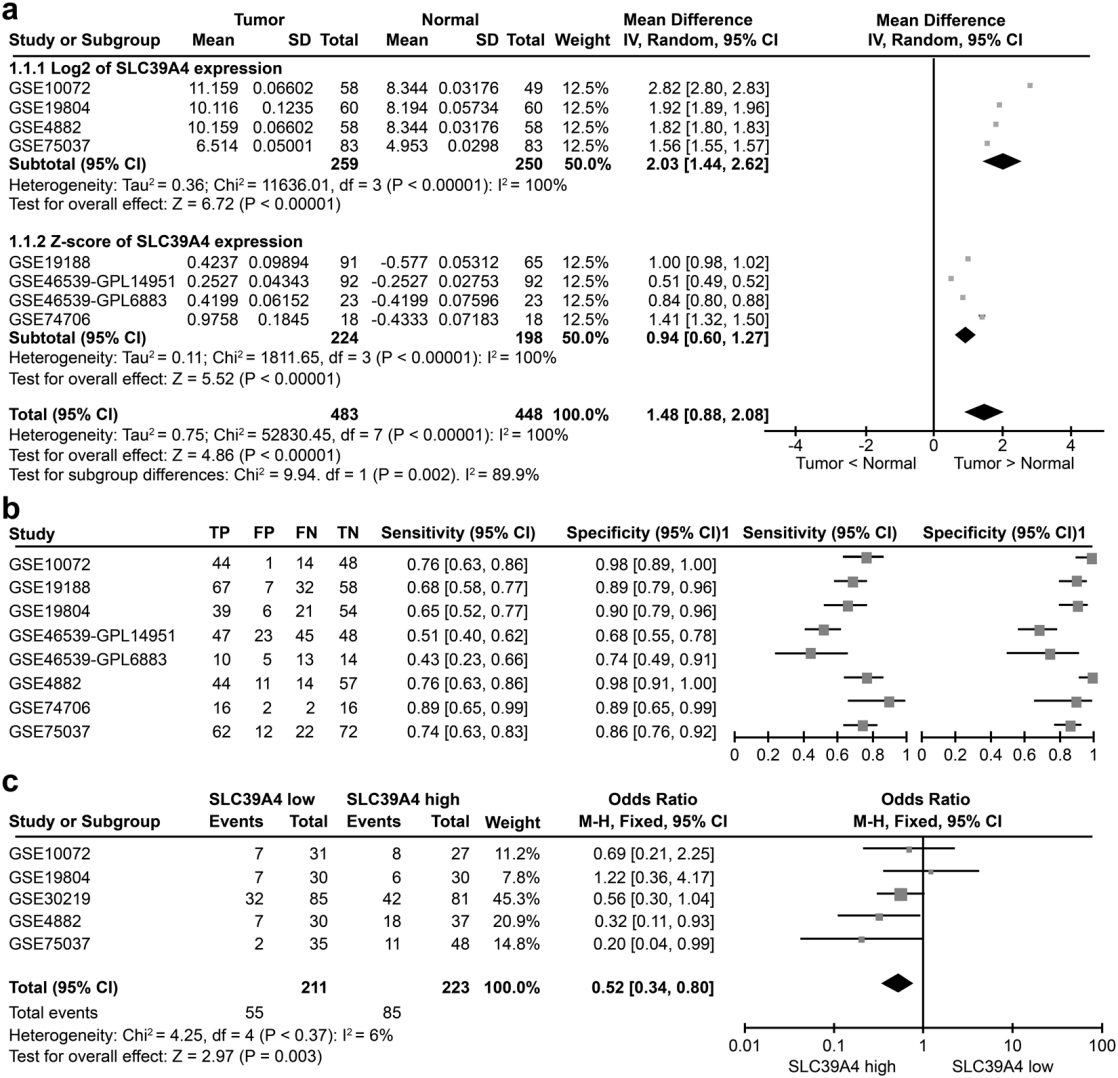
SLC39A4 is an independent predictor of non-small cell lung cancer (NSCLC). (**a**) Meta-analysis of SLC39A4 expression in NSCLC samples and normal tissues. Mean differences were estimated using an inverse variance (IV)-weighted random-effects model (Mean difference, 1.48; 95% CI, 0.88–2.08). (**b**) Meta-analysis of SLC39A4 specificity and sensitivity as a biomarker of NSCLC. (**c**) SLC39A4 expression association with tumour stage using a Mantel-Haenszel (M-H)-weighted random-effects model (Odds Ratio [OR], 0.52; 95% CI, 0.34–0.80).

To study the role and function of SLC39A4 in NSCLC pathogenesis, we first assessed the levels of SLC39A4 expression in normal and lung carcinoma tissues in a meta-analysis of eight GEO datasets containing clinicopathological information (Supplementary Table S1). Notably, we found that SLC39A4A expression was significantly higher in lung cancer samples than in normal lung tissues (*Z* = 4.86, *P* < 0.00001) (Fig. 1a). Furthermore, a meta-analysis of SLC39A4 expression specificity and sensitivity supported its use as a diagnostic indicator (Fig. 1b). As shown in Fig. 1c, SLC39A4 expression is also markedly higher in stage III-IV cancers than in stage I-II counterparts (*Z* = 2.97, *P* < 0.003), indicating that SLC39A4 expression is associated with NSCLC progression.

### SLC39A4 expression is negatively correlated with overall survival (OS) and disease-free survival (DFS) in NSCLC

**Figure 2:**
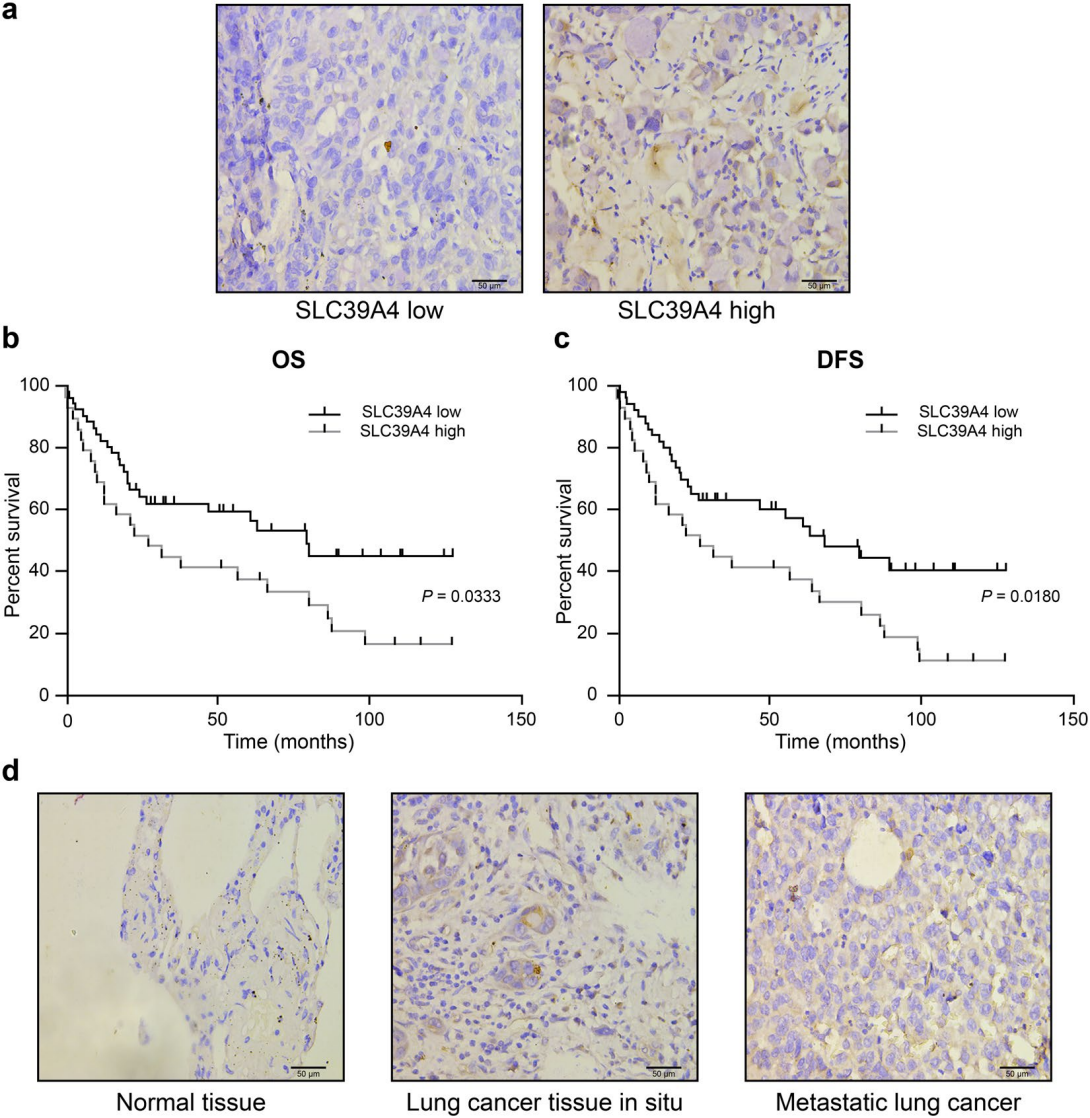
Kaplan-Meier analysis of association of SLC39A4 expression with patient survival. (**a**) Representative images of SLC39A4 low and SLC39A4 high samples (scale bar, 50 μm). (**b**,**c**) Association between SLC39A4 expression and overall survival (OS, **b**) and disease-free survival (DFS, **c**) by Kaplan-Meier analysis. (**d**) SLC39A4 expression in normal tissue, lung cancer *in situ*, and metastatic lesions (scale bar, 50 μm).

We queried the GEO and TCGA databases and found seven datasets that included data on various survival outcomes. A meta-analysis of these studies revealed that increased tumour SLC39A4 expression was associated with shortened OS (*Z* = 2.05, *P* < 0.04) and DFS (*Z* = 2.46, *P* < 0.01) (Supplementary Figure 1a,b). To confirm this finding, we examined the association between SLC39A4 expression and survival outcomes in lung tumour arrays by immunohistochemistry. For this, the 90 tumour samples were dichotomized into groups with high or low SLC39A4 expression based on the median staining intensity observed in the array (μ = 6.516) (Fig. 2a), and then evaluated for OS and DFS by Kaplan-Meier analysis (Fig. 2b,c). Notably, we found that high SLC39A4 expression was a significant indicator of poor OS and DFS in NSCLC (OS: *HR* = 2.918, 95% *CI*: 2.353–3.482, *P* = 0.0333; DFS: *HR* = 2.498, 95% *CI*: 1.924–3.072, *P* = 0.0180), consistent with the results of our meta-analysis. Moreover, we also determined that SLC39A4 expression was associated with increased tumour volume (*P* = 0.0105), regional lymph node spread (*P* = 0.0030), and clinical stage (*P* = 0.0412) (Table 1). More importantly, regional lymph nodes (*HR* = 2.562, *P* = 0.013), clinical stage (*HR* = 2.118, *P* = 0.022) and SLC39A4 overexpression (*HR* = 3.067, *P* = 0.019) were also independent predictors for OS in the multivariate analysis (Table 2). As shown in Fig. 2d, we also observed an increase in SLC39A4 expression corresponding with the progression of *in situ* tumours to metastatic lesions.

**Table 1:** Association of SLC39A4 expression and clinicopathological findings in NSCLC.

Factors	No. of Cases	Average Staining Score ± SD	P-value
Sex			
* Male*	49	6.441 ± 0.2136	0.7848
* Female*	41	6.607 ± 0.5352	
Age			
<*60*	41	6.955 ± 0.3254	0.1967
≥60	49	6.149 ± 0.3868	
Primary tumour (T)			
* T1–T2*	67	5.950 ± 0.3147	0.0105
* T3–T4*	23	8.166 ± 0.5445	
Regional lymph nodes (N)			
* N0–N1*	56	5.412 ± 0.2272	0.0030
* N2–N3*	19	9.771 ± 0.6109	
Clinical stage			
* I–II*	57	5.852 ± 0.2542	0.0412
* III–IV*	29	7.793 ± 0.6199	

Clinical and TNM staging were scored according to the American Joint Committee on Cancer: AJCC Cancer Staging Manual, 7th edition.

**Table 2:** Summary of univariate and multivariate analysis of overall survival in NSCLC.

Parameters	Univariate analysis			Multivariate analysis		
	HR	95% CI	P-value	HR	95% CI	P-value
Sex						
* Male vs. Female*	1.314	0.752–1.972	0.453			
Age						
<*60 vs*. ≥*60*	1.118	0.683–1.593	0.166			
Primary tumour (T)						
* T1–T2 vs. T3–T4*	1.910	0.962–2.872	0.041			
Regional lymph nodes (N)						
* N0–N1 vs. N2–N3*	2.238	1.929–3.682	0.006	2.562	1.992–3.232	0.013
Clinical stage						
* I–II vs. III–IV*	2.109	1.848–3.563	0.031	2.118	1.890–2.441	0.022
SLC39A4 expression						
* Low vs. High*	2.918	2.353–3.482	0.033	3.067	2.190–3.566	0.019

### SLC39A4 silencing inhibits lung cancer cell metastasis *in vitro*

**Figure 3:**
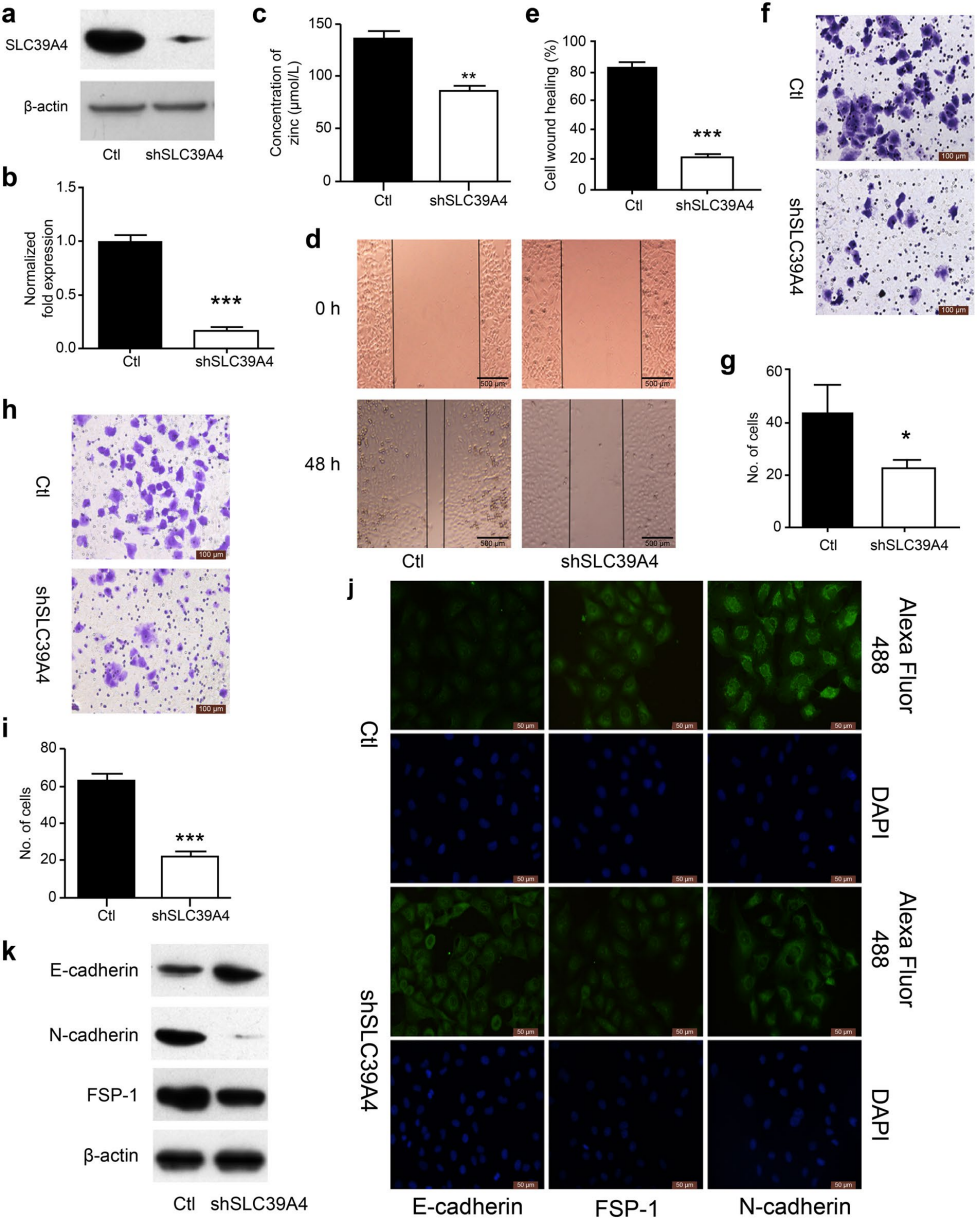
SLC39A4 silencing inhibits A549 cells metastasis *in vitro*. (**a**,**b**) SLC39A4 expression in A549 knockdown and empty vector control (Ctl) cells by western blot analysis (**a**) and qPCR (**b**,**c**). Zn^2+^ concentration in A549 cells after knockdown of SLC39A4 *in vivo*. (**d**,**e**) Analysis of SLC39A4 knockdown and Ctl cell migration in wound-healing assays (scale bar, 500 μm). Representative images (**d**) and quantitation (**e**) are shown. (**f**,**g**) Cell migration was monitored in transwell assays with SLC39A4 knockdown and Ctl A549 cells (scale bar, 100 μm). Representative images (**f**) and quantitation **(g)** are shown. (**h**,**i**) Cell invasion was monitored in matrigel transwell assays with SLC39A4 knockdown and Ctl A549 cells (scale bar, 100 μm). Representative images (**h**) and quantitation (**i**) are shown. (**j**,**k**) Analysis of E-cadherin (epithelial marker) and FSP-1 and N-cadherin (mesenchymal markers) expression in knockdown and control cells by immunofluorescence staining (**j**) and western blotting (**k**) (scale bar, 50 μm).

EMT is as a key step in the metastatic initiation of cancer cells^22^. Because tumour metastasis is significantly detrimental to the survival of patients with lung cancer, we further investigated the effects of SLC39A4 expression on lung cancer cell migration. A549 cells were infected with lentivirus expressing SLC39A4 shRNA or control lentivirus and potent knockdown was confirmed by qPCR and western blot analysis for mRNA and protein expression, respectively (*P* < 0.01; Fig. 3a,b). We found that SLC39A4 knockdown inhibits the Zn^2+^ concentration of cells *in vivo* (Fig. 3c). Wound-healing, transwell, and matrigel invasion assays revealed that SLC39A4 knockdown significantly inhibited A549 cell migration (Fig. 3d–i). Consistently, immunofluorescence and western blot analysis showed a marked upregulation of the epithelial marker E-cadherin accompanied by a concomitant downregulation of the mesenchymal markers FSP-1 and N-cadherin in response to SLC39A4 silencing (Fig. 3j,k). The same experimental results were obtained from SPC-A-1 cells (Supplementary Figure 2). In addition, the function of SLC39A4 in EMT was investigated in normal lung epithelial cell line BEAS-2B; we found that SLC39A4 knockdown reduced the Zn^2+^ concentration and inhibited the expression of EMT makers (Supplementary Figure 3). This pattern was also observed in the GEO meta-analysis, where SLC39A4 levels were negatively correlated with E-cadherin expression (*Z* = 7.60, *P* < 0.00001; Supplementary Figure 4a), but positively associated with FSP-1 (*Z* = 6.56, *P* < 0.00001) and N-cadherin (*Z* = 6.59, *P* < 0.00001; Supplementary Figure 4b,c) expression.

### SLC39A4 silencing attenuates metastatic spread in a mouse model of lung cancer

**Figure 4:**
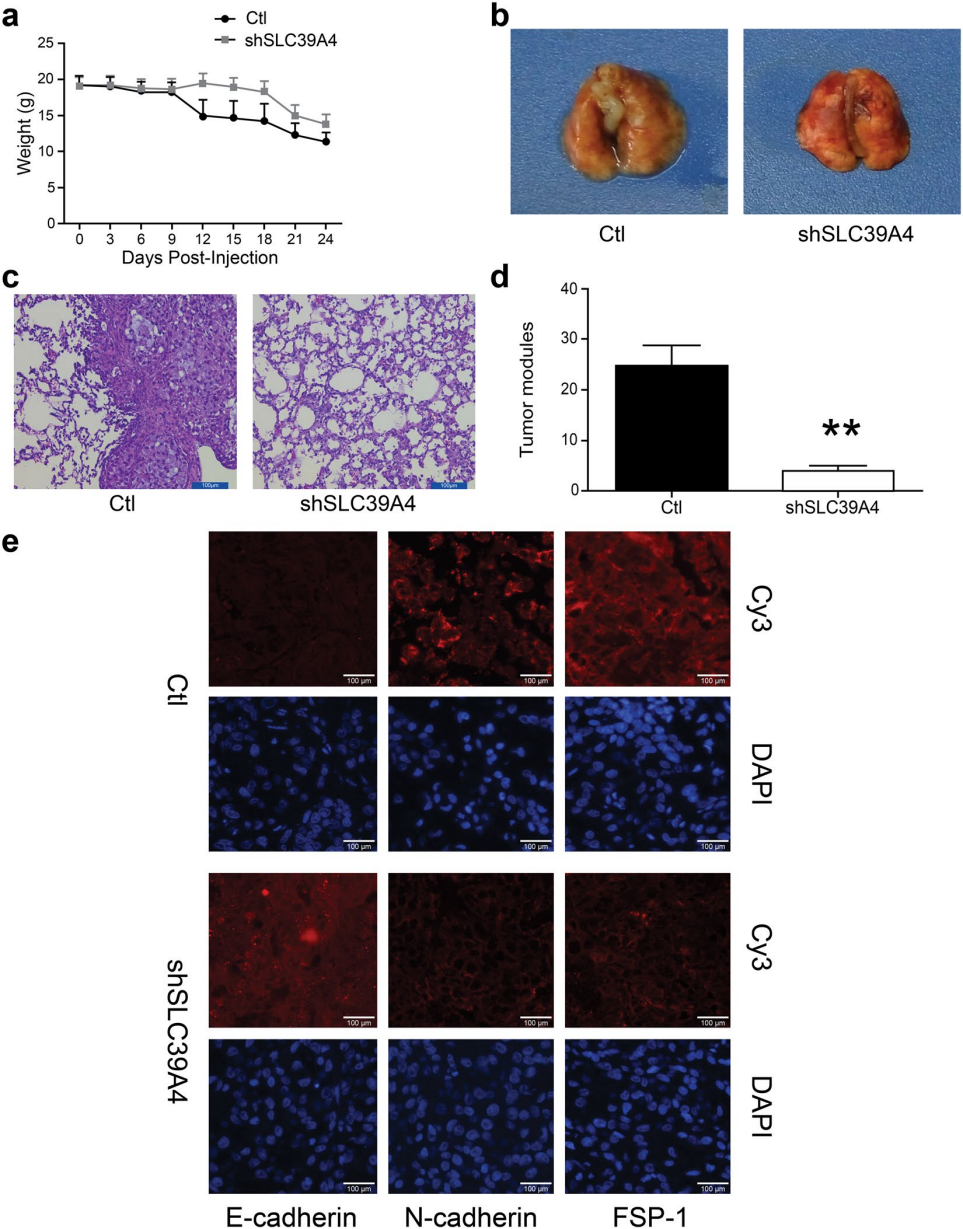
SLC39A4 silencing limits metastatic spread in a mouse model of lung cancer. (**a**) Body weight measurements of tumour-bearing mice. (**b**) Representative images of mouse lungs 30 d after shSLC39A4 or Ctl A549 cell engraftment. (**c**) Representative images of H&E staining in lung tissue sections from each group (scale bar, 100 μm). (**d**) The average number of metastatic tumour nodules in the lungs xenograft mice. E. Immunofluorescence for E-cadherin, FSP-1, and N-cadherin expression in A549 shSLC39A4 or Ctl subcutaneous tumours (scale bar, 100 μm).

We further investigated the metastatic capability of shSLC39A4 cells in mouse model of lung cancer. Notably, mice injected with shSLC39A4 knockdown cells exhibited a smaller loss in body weight (Fig. 4a) that corresponded with diminished pulmonary metastasis when compared to control mice (*P* < 0.01; Fig. 4b–d). Moreover, immunofluorescence of A549 subcutaneous tumours confirmed the increased E-cadherin and decreased FSP-1 and N-cadherin expression in shSLC39A4 A549 cells.

### SLC39A4 silencing promotes sensitivity to cisplatin in human NSCLC cells

**Figure 5:**
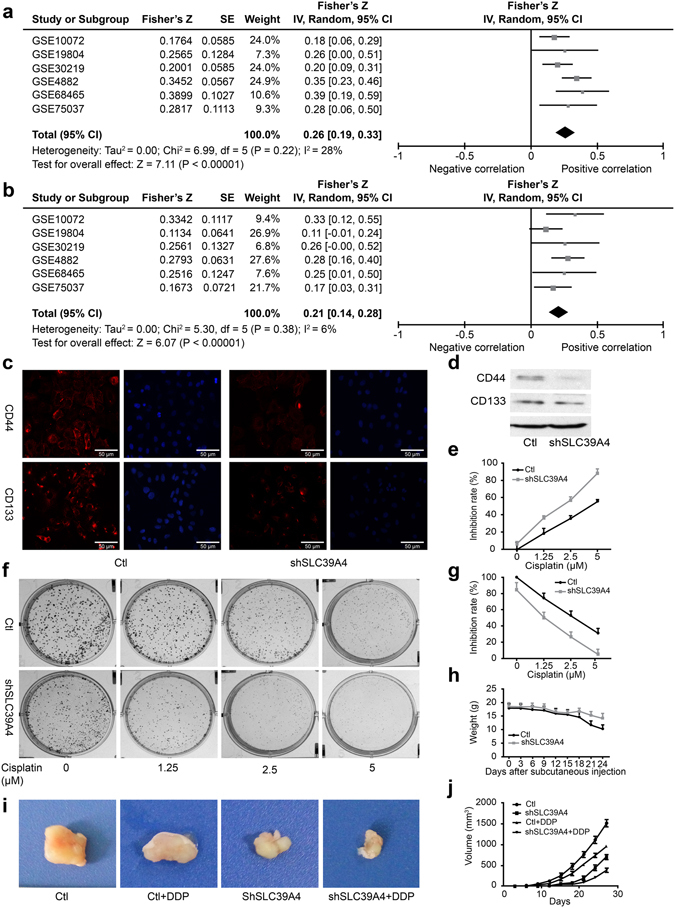
Inhibition of SLC39A4 enhances cisplatin resistance in human NSCLC cells. (**a**) Correlation of SLC39A4 and CD44 expression, Fisher’s Z = 0.26 (95% CI, 0.19–0.33). (**b**) Correlation of SLC39A4 and CD133 expression, Fisher’s Z = 0.21 (95% CI, 0.14–0.28). (**c**,**d**) Analysis of CD44 and CD133 expression by immunofluorescence (**c**) and western blotting (**d**) in shSLC39A4 or Ctl A549 cells (scale bar, 50 μm). (**e**,**f**) Cisplatin-induced cell death was monitored in CCK-8 arrays (**e**) and colony formation assays (**f**,**g**). Quantitation of colony formation. (**h**–**j)** Body weight (**h**) and tumour size (**i,j**) were used to examine cisplatin sensitivity in shSLC39A4 and Ctl A549 tumour-bearing mice.

Our previous results suggested that shSLC39A4 depressed the mesenchymal phenotype of A549 cells. Because many studies have shown that the EMT conveys resistance to radiotherapy or chemotherapy^12^, we speculated that SLC39A4 silencing could affect the chemotherapeutic response by regulating stemness. SLC39A4 expression was significantly correlated with the expression of the lung cancer stem cell biomarkers CD44 and CD133^23^ (*Z* = 7.11, *P* < 0.00001; Fig. 5a,b). This finding was further confirmed by immunofluorescence and western blot analysis of CD44 and CD133 expression in shSLC39A4 and Ctl A549 cells (Fig. 5c,d).

Cancer stem cells are associated with chemoresistance^24^. Cisplatin is the first-line drug for lung cancer therapy; however, resistance is highly prevalent in patients and significantly limits survival. Thus, we further explored the functional significance of SLC39A4 expression in cisplatin resistance. Notably, the CCK-8 assay and colony formation assays revealed that shSLC39A4 sensitized A549 cells to cisplatin-induced cell death (Fig. 5e–j), suggesting that SLC39A4 enhances cellular resistance to cisplatin therapy. Similar findings were also observed in a subcutaneous tumour model with cisplatin treatment, where shSLC39A4 A549 tumours caused less body weight loss (Fig. 5h) and were less proliferative and resistant to cisplatin treatment (Fig. 5i,j).

## Discussion

Lung cancer is the leading cause of cancer-related death in men and women^25^, with more than one million new cases diagnosed each year. Despite recent advances in treatment modalities, including surgery, chemotherapy, and radiotherapy, cancer metastasis remains the major determinant of poor outcome, with an overall 5-year survival rate of 15%^26^. Thus, it is important to discover novel lung cancer metastasis-related genes and their functional mechanisms to encourage the development of additional cancer therapeutics.

The combined use of tumour-related gene expression data and clinical specimens has proven to be a valuable method for identification of potential drug targets^27–29^. Studies have indicated that zinc transport and homeostasis play important roles in cancer progression^30–32^; however, their functional significance in lung cancer is not clear. In this study, a meta-analysis of data extracted from the GEO and TCGA databases revealed the clinical significance of SLC39A4 in lung cancer. Specifically, SLC39A4 expression was markedly higher in NSCLC tissues than in normal controls and was a prognostic indicator of increased metastatic stage, as well as poor OS and DFS. Collectively, these data suggest that SLC39A4 may play an important role in lung cancer pathophysiology.

The EMT plays a significant role during tumour invasion and metastasis^33^, and is characterized by the acquisition of mesenchymal markers such as vimentin or FSP-1, and loss of epithelial cell adhesion molecules, such as E-cadherin^22, 34, 35^. To further study the effect of SLC39A4 on the aggressive cellular characteristics of NSCLC, we infected A549 cells with lentivirus expressing shSLC39A4 or empty vector control. Notably, knockdown of SLC39A4 induced a more epithelial-like phenotype with increased E-cadherin and decreased FSP-1 expression, suggesting that SLC39A4 promotes EMT in A549 cells. The clinical relevance of these results was confirmed with *in vitro* migration assays and a mouse model of tumour metastasis, indicating that SLC39A4 plays an important role in NSCLC cell migration.

Many studies have demonstrated that the EMT facilitates resistance to radiotherapy and chemotherapy^36, 37^. Cisplatin (cis-diamminedichloro-platinum II) is the primary chemotherapeutic agent used in lung cancer therapy, particularly NSCLC^38^. Because acquired resistance is a common occurrence in NSCLC patients^39^, insights into the molecular mechanisms underlying cisplatin resistance is necessary for the development of novel therapeutic strategies. Consistently, SLC39A4 knockdown cells displayed heightened sensitivity to cisplatin-induced cell death when compared to control cells.

In conclusion, the present study demonstrated that SLC39A4 is overexpressed in NSCLC and correlates with increased staging and diminished patient survival. Moreover, silencing of SLC39A4 induced an epithelial-like phenotype, decreased cancer stem cell marker expression, and increased cisplatin sensitivity. Thus, these findings suggest that SLC39A4 may serve as a prognostic biomarker and putative therapeutic target to enhance chemosensitivity in NSCLC.

## Materials and Methods

### Public data analysis

Gene expression data were obtained from the Gene Expression Omnibus (GEO) and The Cancer Genome Atlas (TCGA) databases. The meta-analysis included eight datasets encompassing 942 lung cancer patients (Supplementary Table S1), whereas the prognostic analysis included seven datasets with 1623 total patients (Supplementary Table S2). Raw CEL files were downloaded from the GEO database (http://www. ncbi.nlm.nih.gov/geo/), and background correction and data extraction was performed in R software (R version 3.3.0). The meta-analysis was conducted in Review Manager (RevMan Version 5.3, Copenhagen, Denmark), using a random-effects model because the expression data were acquired by different means. Results are presented in forest plots. Cochran χ2 and *I* ^2^ analyses were performed to assess heterogeneity among the studies involved.

### Reagents

The anti-SLC39A4 antibody was obtained from Abcam (Cambridge, UK). All other antibodies were obtained from Proteintech Group, Inc. (Wuhan, China). Secondary antibodies were purchased from Santa Cruz Biotechnology (Dallas, TX, USA). All other kits and reagents were purchased from the Beyotime Institute of Biotechnology (Shanghai, China). Tissue arrays were from Outdo Biotech Co., Ltd. (Shanghai, China).

### SLC39A4 immunohistochemistry

Tissue arrays were dewaxed and antigens retrieved using high pressure. Endogenous peroxidases were blocked with 3% hydrogen peroxide for 10 min. After immersion in normal goat serum for 30 min, tissues were incubated with the primary antibody at 4 °C overnight, washed with phosphate-buffered saline (PBS), and then incubated with a biotin-conjugated secondary antibody for 30 min at 37 °C. After washing, the sections were incubated with horseradish peroxidase (HRP) complex for 30 min at 37 °C and visualized using diaminobenzidine (DAB). All immunohistochemical images were obtained under an Olympus BX51 microscope equipped with a 20× , a 40× , or 100× objective lens (Olympus, Tokyo, Japan) and a DP 50 camera (Olympus). Images were processed using DPC controller software (Olympus).

Immunohistochemical staining was evaluated by a semiquantitative scoring method. The SLC39A4 staining was scored as follows: no staining (0), light positive staining (1), medium positive staining (2), and strong positive staining (3). The area of positive staining was scored as: <5% (0), 5–25% (1), 26–50% (2), 51–75% (3), and >75% (4). An overall score was calculated by multiplying the intensity and expression scores for each sample. SLC39A4 expression was dichotomized using median staining intensity as the cutoff to define “high” or “low” as above or below the median, respectively.

### Cell culture

The A549 and SPC-A-1 lung cancer cells and the BEAS-2B normal lung epithelial cell line were maintained in RPMI-1640 medium supplemented with 10% (vol/vol) foetal bovine serum (FBS), 10 mM l-glutamine, and 5 mg/mL penicillin/streptomycin at 37 °C with 5% CO_2_. All media and supplements were purchased from Invitrogen (Carlsbad, CA, USA).

### SLC39A4 lentiviral silencing

A549 cells were infected with a lentivirus encoding SLC39A4 shRNA (5′-ACGTAGCACTCTGCGACATGGTCAGGATG-3′) following the manufacturer’s instructions. Briefly, lentiviral particles were purchased from GeneCopoeia (Guangzhou, China) and used to infect A549 cells. Stable cell lines expressing shSLC39A4 or psi-LVRH1MP empty vector (Ctl) were then selected in medium containing 0.5 μg/mL puromycin.

### Detection of Zn^2+^ concentration

Zn^2+^ concentration was measured by atomic absorption spectrometry. After centrifugation, cells were treated with 2% HCL for 1 hour, detection by atomic absorption spectrometer (Beijing Bohui Innovation Technology CO., LTD, BH2100S-5500S).

### Immunofluorescence

Cultured cells were fixed with 4% paraformaldehyde, washed twice with PBS, and then blocked with PBS containing 10% normal goat serum. Cells were then stained with anti-E-cadherin, anti-vimentin, or anti-FSP1 polyclonal antibody for 30 min at 37 °C, washed twice with PBS, stained with Cy3 (red) or Alexa Flour 488 (green)-conjugated secondary antibody for 30 min at 37 °C, and then washed twice again before imaging. All immunofluorescence images were obtained with an Olympus BX51 microscope equipped with a 20 × or 40 × objective lens (Olympus) and a DP 50 camera (Olympus). Images were processed using DPC controller software (Olympus).

### Transwell assays

Cells were cultured in 10-cm plates, and fresh medium was added 18 h before each assay. Cells were trypsinized, washed twice, resuspended in serum-free medium, and then counted using a haemocytometer. RPMI 1640 with 10% serum was added to the lower wells of the transwell chamber apparatus, and 10,000 cells in 200 μL of serum-free media were added to each upper well. The loaded chamber was incubated for 24 h at 37 °C, at which time the cells on the upper membrane surface were removed by scraping to leave only those that had migrated through the membrane. The transwell membranes were then fixed in methanol, stained with 0.1% crystal violet, and air-dried. The number of cells in each field was quantified and presented as an average from five fields of triplicate wells for each test condition.

### Western blotting

Protein samples were resolved by SDS-PAGE on 12% gels and transferred to nitrocellulose membranes, which were then blocked for 1 h at room temperature in Tris-buffered saline (TBS) containing 0.1% Tween 20 and 5% fat-free milk. Primary antibody incubations were performed for 18 h at 4 °C. HRP-conjugated secondary antibody incubations were performed at room temperature for 1 h and visualized with enhanced chemiluminescence (SuperSignal; Pierce, Rockford, IL) or ECL Plus (Amersham Pharmacia Biotech, Buckinghamshire, United Kingdom) substrates according to the manufacturers’ instructions.

### Cell viability assays

Cell viability was assessed by colony formation assays and cell counting kit (CCK)-8 assay. Briefly, cells were plated at 500 cells per well in a 6-well plate (Corning, Corning, NY, USA) after being treated with different concentrations of cisplatin (0, 1.25, 2.5, or 5 μg/mL). Cells were cultured for 10 days with medium changes every 3 days. Colonies were washed with PBS, fixed in methanol, and then stained with crystal violet. The CCK-8 assay was completed according to the manufacturers’ instructions.

### Mouse xenograft model

All xenograft experiments were performed in accordance with the guidelines of the Laboratory Animal Ethical Committee at Chengdu Medical College. All experimental protocols were approved by the Laboratory Animal Ethical Committee at Chengdu Medical College (Approval date: 2017.07.20; Approval number: CYYFYEC2016003). An A549 lung metastasis model was established to investigate the effect of SLC39A4 on cell migration and invasion *in vivo*. Female nude mice (6–8 weeks old, 20–22 g) were purchased from the Experimental Animal Center of Sichuan University (Chengdu, Sichuan Province, China). Briefly, ten nude mice were intravenously inoculated with 5 × 10^5^ A549 cells expressing shSLC39A4 or the empty vector control and euthanized 30 days later to count the pulmonary metastatic nodules. The lungs were fixed in Bouin’s solution after removal. Formalin-fixed, paraffin-embedded sections of each lung tissue sample were stained routinely with H&E.

A subcutaneous A549 model was established to investigate the effect of SLC39A4 on cisplatin treatment and EMT *in vivo*. Ten nude mice were subcutaneously inoculated with 5 × 10^5^ A549 cells expressing shSLC39A4 or an empty vector control. The tumour samples were fixed in Bouin’s solution after removal. Formalin-fixed, paraffin-embedded sections of each sample were stained routinely with EMT biomarkers (E-cadherin, N-cadherin, and FSP-1). Histopathological examination and immunofluorescence imaging were completed under an Olympus microscope.

### Statistical analysis

Each experiment was performed at least three times independently. The association of SLC39A4 expression with clinicopathological parameters was analysed by paired t-test or one-way ANOVA in GraphPad Prism 5 (GraphPad, San Diego, CA, USA). Statistical significance was defined as *P* < 0.05.

## Electronic supplementary material

Supplementary Material
