# Supplementary material for: SLC39A4 expression is associated with enhanced cell migration, cisplatin resistance, and poor survival in non-small cell lung cancer
Source: Sci Rep. 2017 Aug 3;7:7211. doi: 10.1038/s41598-017-07830-4 (PMC5543149; doi:10.1038/s41598-017-07830-4)
Supplement: Supplementary file 1 — Supplementary Material [file 41598_2017_7830_MOESM1_ESM.doc]

**SLC39A4 expression is associated with enhanced cell migration, cisplatin resistance, and poor survival in non-small cell lung cancer**

Dong-ming Wu, Teng Liu, Shi-hua Deng, Rong Han, Ying Xu*

Clinical Laboratory, The First Affiliated Hospital of Chengdu Medical College, Chengdu, Sichuan 610041, P.R. China

* Corresponding author. e-mail address: Yingxu825@126.com

**Supplementary Tables**

Supplementary Table S1. Study information on eight GEO datasets that consist of normal lung and lung cancer samples.

| **GEO Series** | **Country** | **Normal** | **Lung Cancer** | **Platform** | **Analysis Method** |
| --- | --- | --- | --- | --- | --- |
| GSE10072 | USA | 49 | 58 | GPL96 | Array |
| GSE19188 | Netherlands | 65 | 99 | GPL570 | Array |
| GSE19804 | Taiwan | 60 | 60 | GPL570 | Array |
| GSE46539-GPL6883 | Taiwan | 92 | 92 | GPL6883 | Array |
| GSE46539-GPL14951 | Taiwan | 23 | 23 | GPL14951 | Array |
| GSE4882 | Taiwan | 58 | 58 | GPL3730 | Array |
| GSE74706 | Germany | 18 | 18 | GPL13497 | Array |
| GSE75037 | USA | 84 | 84 | GPL6884 | Array |
| Total |  | 449 | 492 |  |  |

**Supplementary Table S2. Information on four GEO datasets and three TCGA cancer studies that consist of lung cancer samples with OS and DFS data.**

| **Study** | **Country** | **Lung Cancer** | **Platform** | **Analysis Method** |
| --- | --- | --- | --- | --- |
| GSE19188 | Netherlands | 82 | GPL570 | Array |
| GSE30219 | France | 293 | GPL570 | Array |
| GSE37745 | Sweden | 196 | GPL570 | Array |
| GSE50081 | Canada | 175 | GPL570 | Array |
| Lung AD (TCGA, Nature 2014) | USA | 203 |  | RNA Seq |
| Lung AD (TCGA, Provisional) | USA | 431 |  | RNA Seq |
| Lung SCC (TCGA, Provisional) | USA | 494 |  | RNA Seq |
| Total |  | 1623 |  |  |

**Supplementary Figures**


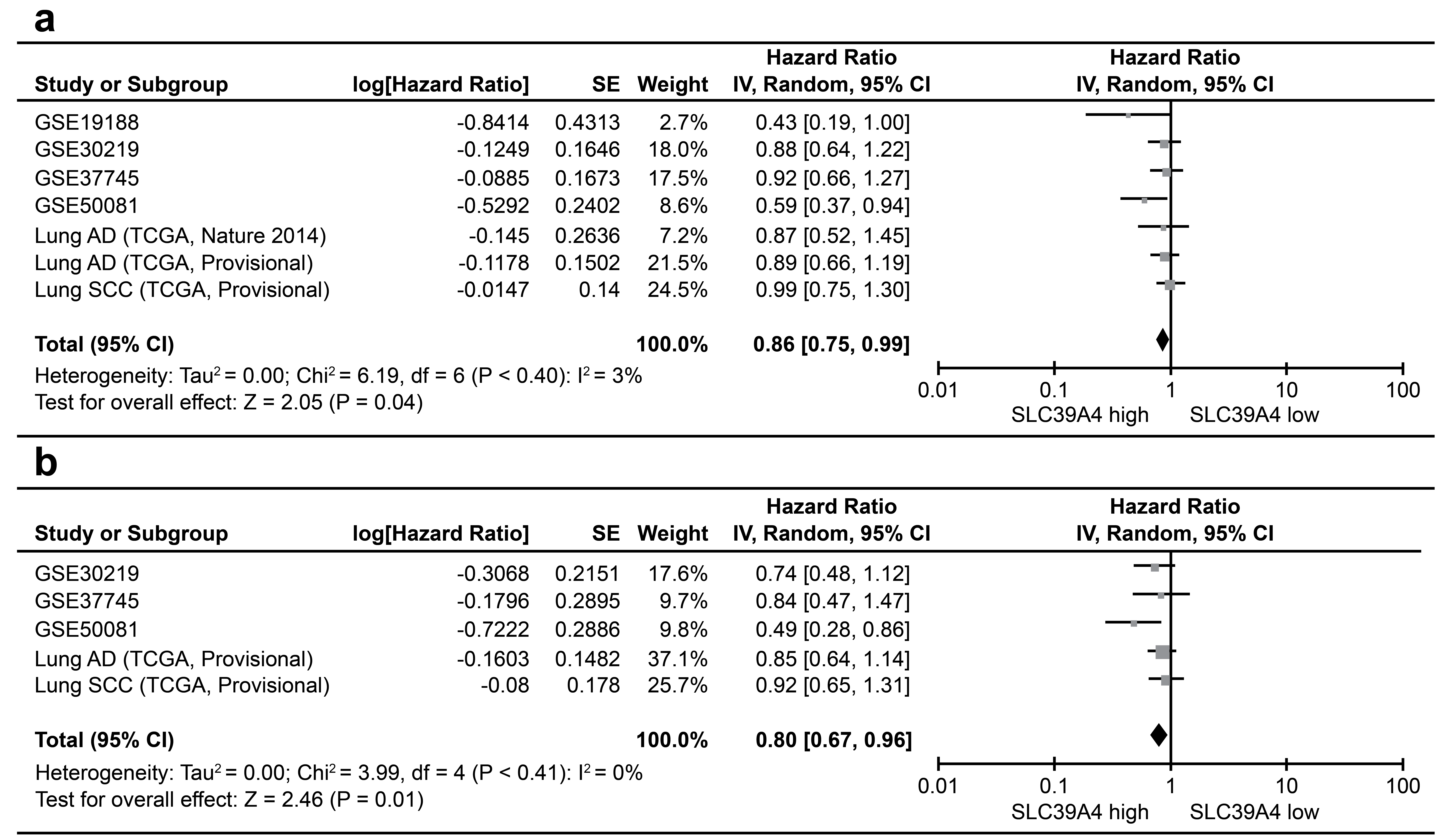


**Supplementary Figure 1. Meta-analysis of the associations between SLC39A4 expression and patient survival in NSCLC.**

**a.** Overall survival (OS): HR = 0.86 (95% CI, 0.97–0.99) for high and low SLC39A4 expression. **b.** Disease-free Survival (DFS): HR = 0.80 (95% CI, 0.67–0.96) between high and low SLC39A4 expression.

**
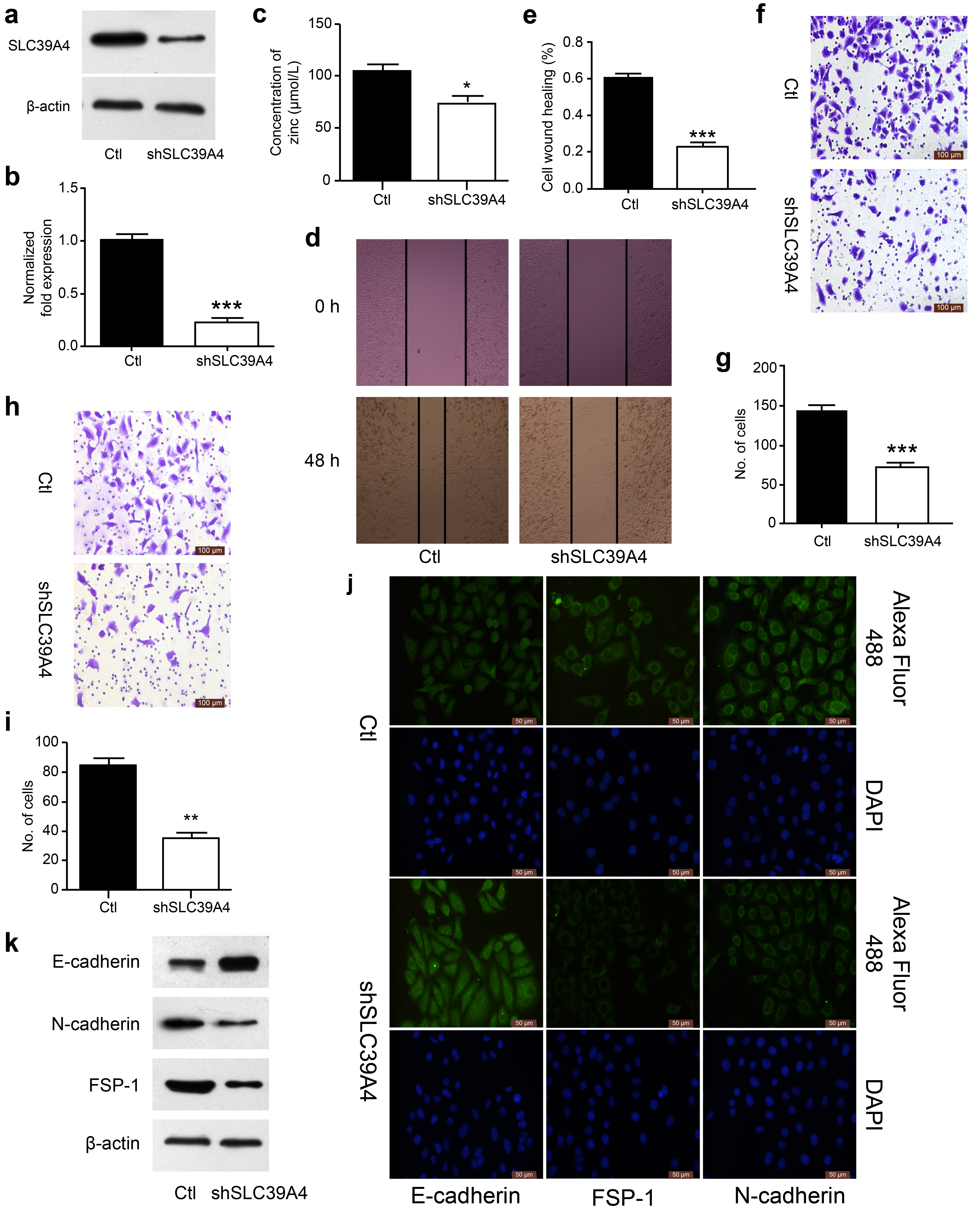
**

**Supplementary Figure 2. SLC39A4 silencing inhibits SPC-A-1 cells metastasis in vitro.**

**a,b.** SLC39A4 expression in SPC-A-1 knockdown and empty vector control (Ctl) cells by western blot analysis (**a**) and qPCR (**b). c.** Zn^2+^ concentration of BEAS-2B cells after knockdown of SLC39A4 in *vivo*. **d,e.** Analysis of SLC39A4 knockdown and Ctl cell migration in wound-healing assays (scale bar, 500 μm). Representative images **(d)** and quantitation **(e)** are shown. **f,g.** Cell migration was monitored in transwell assays with SLC39A4 knockdown and Ctl SPC-A-1 cells (scale bar, 100 μm). Representative images (**f**) and quantitation **(g)** are shown. **h,i.** Cell invasion was monitored via matrigel transwell assays with SLC39A4 knockdown and Ctl SPC-A-1 cells (scale bar, 100 μm). Representative images (**h**) and quantitation **(i)** are shown. **j,k.** Analysis of E-cadherin (epithelial marker) and FSP-1 and N-cadherin (mesenchymal markers) expression in knockdown and control cells by immunofluorescence staining (**j**) and western blotting **(k)** (scale bar, 50 μm)**.**

**
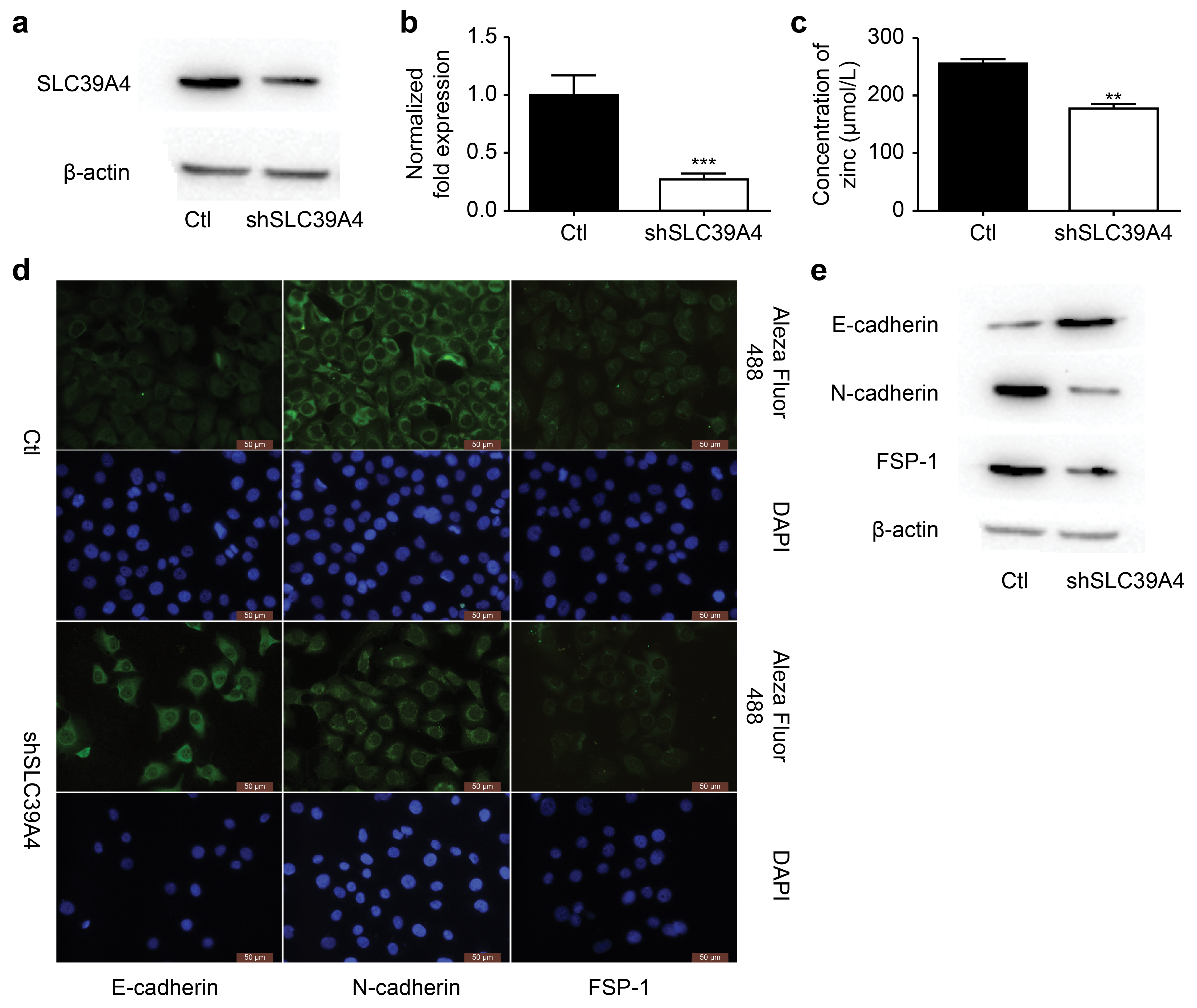
**

**Supplementary Figure 3. SLC39A4 silencing inhibits EMT of BEAS-2B cells in vitro.**

**a,b.** SLC39A4 expression in BEAS-2B knockdown and empty vector control (Ctl) cells by western blot analysis (**a**) and qPCR (**b). c.** Zn^2+^ concentration of BEAS-2B cells after knockdown of SLC39A4 *in* *vivo*. **d,e.** Analysis of E-cadherin (epithelial marker) and FSP-1 and N-cadherin (mesenchymal markers) expression in knockdown and control cells by immunofluorescence staining (**d**) and western blotting **(e)** (scale bar, 50 μm)**.**

**
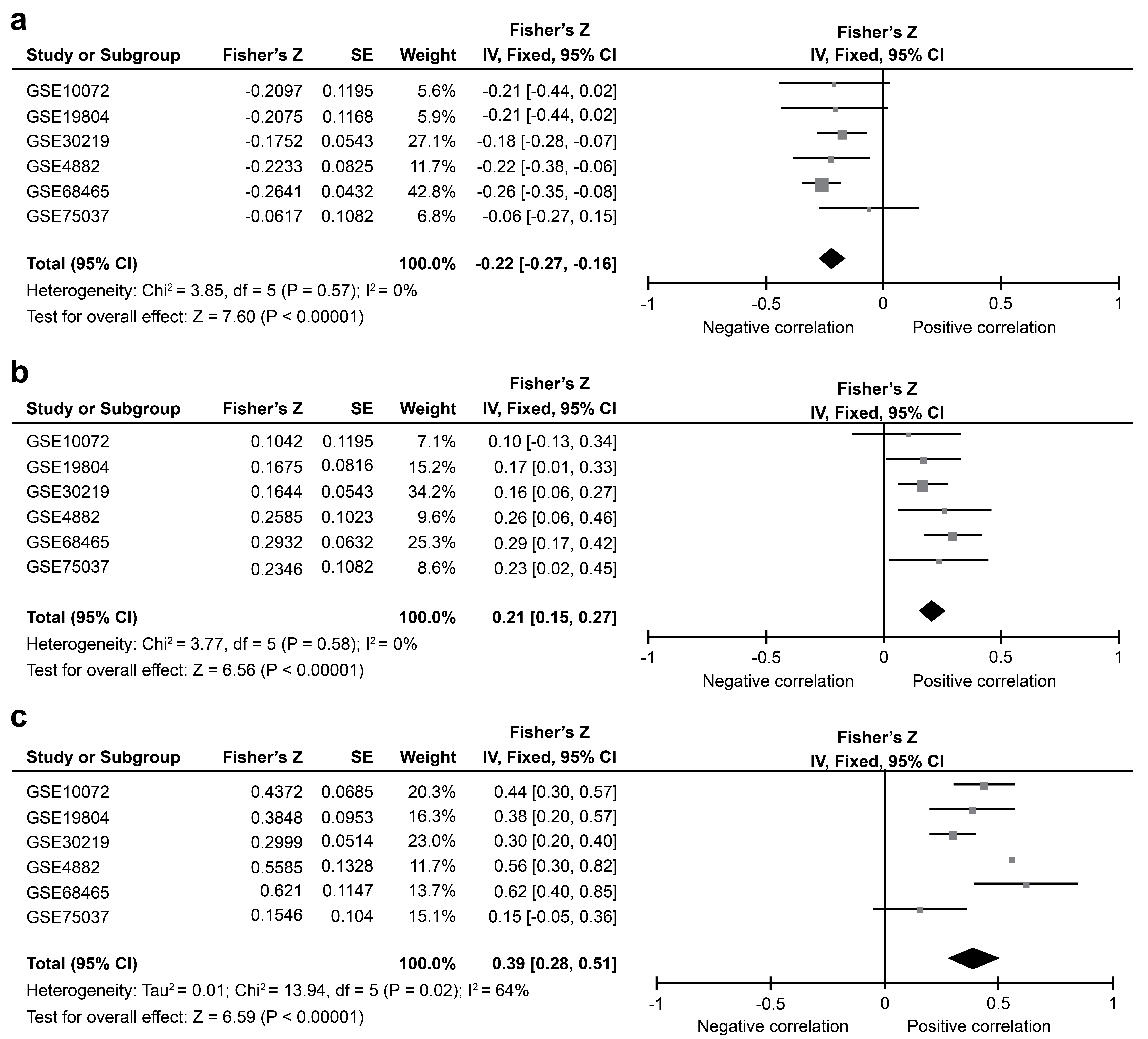
**

**Supplementary Figure 4. Meta-analysis of the associations between of SLC39A4 and E-cadherin, FSP-1, or N-cadherin expression.**

**a.** Correlation of SLC39A4 with E-cadherin: Fisher's Z = -0.22 (95% CI, -0.27–-0.16). **b.** Correlation of SLC39A4 with FSP-1: Fisher's Z = 0.21 (95% CI, 0.15–0.27). **c.** Correlation of SLC39A4 with N-cadherin: Fisher's Z = 0.39 (95% CI, 0.28–0.51).
